# Women’s experiences of rectovaginal fistula: an ethno- religious experience

**DOI:** 10.1186/s12905-020-00992-w

**Published:** 2020-06-19

**Authors:** Fatemeh Touhidi Nezhad, Rostam Jalali, Fozieh Karimi

**Affiliations:** 1 Students’ Research Committee, Kermanshah University of Medical Sciences, Kermanshah, Iran; 2 Kermanshah University of Medical Sciences, Kermanshah, Iran

**Keywords:** Rectovaginal fistula, Phenomenology, Qualitative study, Lived experience

## Abstract

**Background:**

Obstetric fistulas are one of the most tragic injuries that occur after difficult, prolonged childbirth without timely intervention. These fistulas cause discomfort to patients and result in emotional, social, and even physical suffering. The present study aimed to explore the experiences of women with rectovaginal fistula in Kamyaran city, in Kurdestan province, west of Iran.

**Methods:**

In a phenomenological study, 16 patients, healthcare personnel, and patients’ families were investigated. Purposive sampling was performed and Study participants were interviewed in-depth semi-structured interviews. All interviews were audio-recorded, transcribed verbatim (word by word), and analyzed by Colaizzi’s method. For determining the validity of the study, Lincoln and Guba’s criteria, which include credibility, dependability, transferability, and confirmability, were considered.

**Results:**

Five general themes and 10 sub-themes emerged after investigating interviews. Themes include religious harassment the sub-theme of being defiled), fail (sub-themes of loss and negative attitudes, disrupted sex (the sub-theme of sexual dissatisfaction), consequence (three sub-themes of sleep disturbance, mental crisis, and isolation), and ultimately panic (three sub-themes of humiliation, secrecy, and fear).

**Conclusion:**

The rectovaginal fistula is a complex and multifaceted problem with social, individual, familial, religious, and ethnic-environmental dimensions, so there is no simple solution to interact with this problem and there is a need to find a solution, considering the dimensions of the problem and plan for help these patients cope with their disease, and take steps to fully treat it.

## Background

Maternal health refers to the health of mothers during pregnancy, childbirth, and postpartum childbirth. Each mother who wishes to have a baby, wants to have safe childbirth, care, and support during and after childbirth [1]. Obstetric fistulas are one of the most tragic injuries that occur after difficult, prolonged childbirth without timely intervention, and this fistula is a duct between the vagina and the bladder or rectum, which frequently causes urinary and fecal incontinence [2]. These fistulas can also be seen following sexual trauma, especially in the younger sex group [3]. Although eradicated in industrialized countries, this complication continues in low-income countries, affecting poor and vulnerable women [4]. The World Health Organization (WHO) estimates that 50,000 to 100,000 new cases of obstetric fistula occur each year and there are more than 2 million women with fistulas in sub-Saharan Africa and South Asia [5, 6]. These fistulas cause discomfort to patients and result in emotional, social, and even physical suffering [7–10]. In addition to such suffering, these patients are often rejected by their community and husbands and have poor health [10, 11]. Consequently, depression and psychological complications are the consequence of the disease [8, 10, 12], and the problems somewhat persist even with this complication is repaired [13, 14]. The obstetric fistulas have multiple effects as well as medical and psychosocial outcomes, and urinary and fecal incontinence makes it difficult to maintain proper health for individuals and to perform routine social and occupational activities [15]. Lack of awareness and knowledge of the cause and treatment of fistulas by family and community members may lead to misconceptions that may then expose these women to greater stress and stigma, making their overall quality of life very poor and unbearable [4, 14, 16, 17]. Few studies have been conducted on the social consequences and structure of the society in which these women live; on the other hand, although there is a large number of these patients in Iran, there has been no qualitative study determining these women’s experiences, especially in areas of Kurdish culture. Therefore, the present study aimed to explore the experiences of women with rectovaginal fistula in Kamyaran city so that care decision-makers support and manage these patients and make appropriate interventions by understanding the experiences of these patients.

## Methods

The present study was conducted in the first 8 months of 2019. The timing of this problem in women has not been determined, but women in the study have reported problems for more than 5 months. When the study was approved by Kermanshah University of Medical Sciences Ethics Committee, 16 patients, healthcare personnel, and patients’ families were investigated using a descriptive phenomenology and Colaizzi’s method. This method allows the researcher to express the meaning and nature of phenomena in their language and to explore their understanding of the phenomenon. In this approach, the perception of each person is considered as a unique person, but the sum of them contributes to achieving a better understanding of the phenomenon.

### Participants

Purposive sampling was performed on 16 participants, including 14 patients, one midwife, and one family member (mother) in Kamyaran (western Iran). Study participants were coordinated for interviewing after obtaining written informed consent at their preferred location (often a clinic consultation room). Participants have known samples of rectovaginal fistula who referred to the clinic for follow-up treatment, could speak Persian or Kurdish Languages, and completed the consent form. The sample size was determined based on data saturation criteria so sampling continued until data saturation and the emergence of no new code [18]. The presence of a midwife and a patient’s family members were present to maximize variability among participants.

### Data collection

The in-depth semi-structured interviews were used to collect data. In-depth interview used to explore and clarify the meaning of phenomena under study. The order of questions were related to participants’ responses [19]. Before starting the interview, attempts were made to establish communication using general questions. Interviews were conducted in a quiet and private environment, with an average interview duration of 50 min. Participants’ permission over recording the interviews was obtained, and they were assured that the interviews would remain confidential and won’t be used except for the research and that the names and profiles of the participants would remain confidential. Interviews focused on women’s experiences of living with rectovaginal fistula, and the following questions were asked:

- What is your experience with living with a rectovaginal fistula?
- What does a rectovaginal fistula mean to you?
- What is your understanding of living with a rectovaginal fistula?
- How others (family, community) reach your illness)?
- How do you cope with the rectovaginal fistula?

It should be noted that all patients spoke Kurdish.

### Analysis

All interviews were audio-recorded, transcribed verbatim (word by word), and translated to English, where applicable. Pseudonyms were used to maintain anonymity. Data analysis was guided by that described by Colaizzi’s method. Two researchers examined all transcripts for accuracy and completeness against the original notes before data was ready for coding.

The Colaizzi’s seven steps were followed step by step. They included: familiarizing, identifying significant statements, formulating meanings, clustering themes, developing an exhaustive description, producing the fundamental structure, and seeking verification of the fundamental structure [20]. Finally, to validate the findings, there was one face-to-face interview session with some participants being asked some questions about the results. When results were confirmed by the participants, the findings were verified. Several steps were taken for establishing trustworthiness. For determining the validity of the study, Lincoln and Guba’s criteria, which include credibility, dependability, transferability, and confirmability, were considered [21]. To this end, the first author visited all the patients in the local clinic several times. She assessed and examined them routinely. The coding process was controlled by senior qualitative research experts. Also, the process of coding and analyzing the data were described in detail. The themes and sub-themes were sent back to participants in order to confirm the accuracy of the data.

## Results

A total of 14 patients with rectovaginal fistula participated in the study, and the mother of one patient and one treatment staff (master of obstetrics) were interviewed to ensure maximum variability. Most participants had a low educational level, and all were housewives. The most important cause was difficult childbirth and sex. The age range was between 24 to 71 years (Table 1).

**Table 1:** demographic characteristic of participants

Participant no	Marriage	Job	Education level	Cause of fistula	Number of children
1	Married	Housewife	Secondary	First sexual intercourse	0
2	Married	Housewife	Primary	prolonged labor	3
3	Married	Housewife	Primary	prolonged labor	2
4	Married	Housewife	Illiterate	History of colon surgery	9
5	Married	Housewife	Illiterate	History of hysterectomy	4
6	Married	Housewife	Primary	First sexual intercourse	2
7	Married	Housewife	Secondary	History of colon surgery	3
8	Married	Housewife	Secondary	First sexual intercourse	1
9	Married	Housewife	Secondary	prolonged labor	2
10	Married	Housewife	Primary	prolonged labor	1
11	Married	Housewife	Secondary	prolonged labor	2
12	Married	Housewife	Secondary	First sexual intercourse	3
13	Married	Housewife	Primary	prolonged labor	1
14	Separated	Housewife	Secondary	First sexual intercourse	2

Five general themes and 10 sub-themes emerged after investigating interviews (Table 2).

**Table 2:** Concepts and categories extracted from the experiences of women with rectovaginal fistula

Themes	Sub-themes	Codes
Religious harassment	being defiled	Fear of invalidating ablution
		Fear of invalidating prayer
		Fear of not praying
		Fear of defiling the mosque
Fail	Loss	Not pleasant
		To be destroyed
		Being terrible
	Negative attitude	Feel the change of life
		No hope for recovery
		Distrust of the doctor
Disrupted sex	Sexual dissatisfaction	Decrease sex frequency
		Escaping sex
		Making excuses for not having sex
		Having stress during sex
Consequences	Sleep disorder	Sleeping late
		No having deep sleep
	Mental crisis	Fatigue
		Getting angry
		Having pressure
		Being bored
		Feeling bad
	Isolation	Having an impact on the family
		Instability in life
		Escaping parties
		Decreased communication
		Fear of being in the public
Panic	Humiliation	To be ridiculed
		Feeling embarrassed
	Secrecy	Hiding the problem
		Fear of raising the problem
		Hard to explain the problem
	Fear	Always worried
		Fear of eating enough food
		Fear of disgrace
		Always thinking of being in trouble
		Permanent fear of bad smell

Themes include religious harassment (the sub-theme of being defiled), fail (sub-themes of loss and negative attitudes, disrupted sex (the sub-theme of sexual dissatisfaction), consequence (three sub-themes of sleep disturbance, mental crisis, and isolation), and ultimately panic (three sub-themes of humiliation, secrecy, and fear).

One of the main themes was the religious harassment theme. Study participants had difficulty performing their religious duties due to their illness, so they blamed themselves religiously and were not prepared to perform religious activities.

“... Only since I have no control over my urine, defecation and, gas, my Wuzoo doesn’t remain intact for a long time. While I’m praying, I’m always afraid that it breaks my prayer....” (Participant 3).

Or they were afraid of polluting religious sites. “I do not go to the mosque because I am afraid to defile the mosque” (participant 11).

Another important theme was to fail, which included two sub-themes of loss and negative attitude. Study participants often regarded the illness as the loss of everything and saw the future as bleak.

In this regard, one participant said, “To be honest, the disease destroyed my life... let alone lacking control over your urine, defecation, and gas” (Participant 2).

“It’s very bad to be suffering from the filthiest thing. I am very angry in front of my eyes woe betide others and I pray that it will be treated” (Participant 13).

Some participants also regarded the disease as an unpleasant experience and were sometimes desperate. “I have nothing to say but well everything to me was contrary to my dreams and wishes up to this point. Only the first few years of my life were good and financial problems would not allow me to touch happiness at that time, but I wish people did not have all the pain at the same time” (Participant 5).

Another main theme of this study was disrupted sex. Participants were sometimes dissatisfied with their sex, and sometimes escaping it.

“Well, the relationship is a two-way thing, and I must be content with it, but (he) always does its job and doesn’t care about anything” (Participant 7).

“Both my husband and I hate sex” (Participant 9).

“In the early course of the disease, as soon as my husband suggested me to have sex, I said that I’m on my period and have spots, and then I fought with him without planning. Now I have no sex with him once a month” (Participant 14).

Another main theme referred to by participants was panic and fear. Constant humiliation, secrecy, and fear are part of their lives. They always think no to be humiliated and ridiculed, and their name dragged through the mud because of the current situation.

“But my husband mocked me for expelling gas during sleeping a couple of times” (Participant 1).

“I’m so embarrassed to fart, especially my children are boys” (Participant 6).

“What can I say to my family, it’s hard for me to explain a bit” (Participant 4).

“I’m not always worried that my daughter or my son will notice this” (Participant 2).

Another main theme of this study was the consequence. The consequence of this complication for patients in this study was isolation, sleep disturbances, and mental crises. Patients often fall asleep later than others, lest they expel gas at bedtime, in addition to being a light sleeper so that I can manage defecation if it happens. The consequences of the disease often made them nervous and were in a state of mental crisis. On the other hand, these patients have cut family relationships and have often been isolated.

“At night, I always let my husband sleep, then I sleep, and I sleep after making sure he has slept” (Participant 1).

“I try to control as far as I can, but I’m not satisfied. I’m tired of every single second of my life” (Participant 12).

“I especially have to stay in the bathroom for a long time and get angry” (Participant 7).

“And I always try to squeeze my legs if I’m standing next to somebody. Interestingly, the pressure continues until it’s expelled” (Participant 4).

“I reached the stage between hope and hopelessness” (Participant 1).

“It has a big impact on my commuting. I used to go to the village for a week and stayed at my mom’s house, but I don’t want to go now, and I feel like I’m in touch less frequently” (Participant 10).

“But I’m scared to be in the public, and that fear has caused me living in a small family facing some difficulty in daily commute” (Participant 8).

## Discussion

The results showed that the study participants were religiously harassed, are always in a state of fear and anxiety, and in addition to suffering from disrupted sexual health, reached a stage of despair and helplessness, and the disease consequences led them to isolation and mental crisis. Similar studies in different cultures have had similar narratives.

Women with rectovaginal fistula are unable to express themselves. They are in different Ethno- Religious conditions because they feel ashamed and have been suffering from this problem for many years.

Religious harassment was caused by disease interfering with the practice of religion, and unlike the Boscaglia’s study, which regarded religion and spirituality as a solution to women’s disease management [22], the disease was a barrier to religious practices and caused suffering in them. In other studies, the disease has sometimes been regarded as a punishment by God [23]. In contrast, some studies have viewed religion as a way of coping with diseases and have identified a lack of coping as a sign of depression and cognitive impairment [24, 25]. It seems that relying on religion for disease management, as well as interfering with religious practices can have positive and negative consequences; however, overall religion’s benefits are greater and can be used for disease management.

Religion has a positive effect on emotions, hence it is used for managing diseases and coping with stress. Religion provides ways to conduct people’s lifestyles and communicate in social groups. If they follow religion rules, its’ consequences would reduce stress and vice versa can increase strain. Religious beliefs may cause desertion from familial problems as a result of involving in religious activities [26].

Sexual dysfunction in patients with fistula is a major concern and has been discussed in various studies, although the present study emphasized sexual dissatisfaction, pointed to escaping sex, and having stress during intercourse. In another study, escaping sex is called fear of the future lacking a partner, not getting married, and not getting pregnant were among concerns of the patients [11]. Lack of close contact with the husband has been cited as a serious and painful problem [27], and efforts to maintain family and marital relationships have been regarded as an important issue in a systematic review and qualitative meta-synthesis [28]. Women are concerned about the fact that their husband sees them defecating during sexual intercourse [15], which even leads to the fear of divorce and remarriage by the husband [29].

Patients with fistulas often think that their lives have come to an end and regard it as a failure. Having a negative attitude and feeling worthless bother them. The combination of the constant presence of problem and loss of role leads to feelings of worthlessness [30], and women lose self-confidence [15], and the combination of these negative attitudes, physical symptoms, and the reactions of relatives result in these consequences in these patients. These consequences manifest in the form of mental crises, sleep disturbances, and social isolation. All consequences, except for sleep disorders, have been also expressed in similar studies such as sadness, depression, and social isolation [11, 27, 30].

Patients were in constant fear, and none of the patients in the present study disclosed their disease to non-family members and found it necessary to keep it a secret, which has been confirmed by various studies. This has led to psychological consequences in these patients [11, 15, 27, 28, 30, 31].

Although this phenomenological study seeks to explore the meaning of the phenomenon from the participants’ point of view, variables such as the cause of fistula formation, and social life require more attention. Marriage at an early age, especially in rural areas and lack of proper sexual education, exposes young girls to the rectovaginal fistula.

Women getting married at early ages is related to several factors and can deprive them from achieving proper education, attaining a suitable job, and engaging in social relationships or activities [32, 33].

Another cause of rectovaginal fistula is lengthy labour. Over 85% of rectovaginal fistula cases are caused by lengthy obstructed labour in developing countries. A rectovaginal fistula is attributed to obstructed and/or prolonged labour coupled with poor health-seeking behaviours, poor health referral systems, lack of awareness, poor transportation systems, lack of skillful birth attendants, and insufficient obstetric care services. As a result, the survivors of these complications may develop Rectovaginal fistula [29, 34].

Sexuality is an important component of women’s overall well-being, with quality of life and sexual dysfunction contributing to personal and interpersonal stress; this could be especially important for women with genital tract injuries from either obstetric trauma or surgical interventions as one of the major causes of rectovaginal fistula [35]. Given that patient mental health and social functioning appear to improve following surgical fistula repair, patients’ needs to be referred for surgery on time following obstructed labor [13].

Based on the causes of the rectovaginal fistula, steps can be taken to prevent the status. Hence, there are numerous causes for it, so it is possible to improve the situation with a comprehensive approach. This approach include taking place laws for marriage age over 18 years of old, sexual education for girls, better access to health facilities, surgery by expert and skillful midwife, improved referral system and improve access to the health care system.

## Conclusion

The rectovaginal fistula is a complex and multifaceted problem with social, individual, familial, religious, and ethnic-environmental dimensions, so there is no simple solution to interact with this problem and there is a need to find a solution, considering the dimensions of the problem and plan for help these patients cope with their disease, and take steps to fully treat it. Moreover, we should pay attention to the risk factor of this disorder and prevent early marriage while improving health facilities to prevent problems.

## Data Availability

Datasets are available through the corresponding author upon reasonable request.

## Abbreviation

WHO: World Health Organization
